# Sumoylation of Human Translationally Controlled Tumor Protein Is Important for Its Nuclear Transport

**DOI:** 10.1155/2012/831940

**Published:** 2012-04-11

**Authors:** Gnanasekar Munirathinam, Kalyanasundaram Ramaswamy

**Affiliations:** Department of Biomedical Sciences, College of Medicine, University of Illinois, Rockford, IL 61107, USA

## Abstract

Translationally controlled tumor protein (TCTP) lacks nuclear bipartite localization signal sequence; yet TCTP is present abundantly in the nucleus. At present it is not known how TCTP gets transported to the nucleus. Sequence analyses showed that all TCTPs described to date have putative small ubiquitin-like modifier (SUMO) motifs. Since SUMO modification plays an important role in the nuclear transport of proteins, we evaluated whether SUMO motifs are important for transport of TCTP into the nucleus. We show that TCTP exists in sumoylated form in cytoplasm and nucleus of mammalian cells. Point mutation of lysine residue in the SUMO motif compromised the ability of TCTP to get sumoylated *in vitro*. When cells were transfected with FLAG-tagged mutated TCTP, nuclear transport of TCTP was inhibited confirming that sumoylation is critical for the nuclear transport of TCTP. Our previous studies demonstrated that TCTP can function as an antioxidant protein in the nucleus. When we mutated TCTP at the SUMO motif the antioxidant function of TCTP was compromised. Results presented in this study thus show that sumoylation plays an important role in the transport of TCTP into the nucleus where they function as antioxidant protein.

## 1. Introduction

TCTP is a growth-associated protein ubiquitously present in wide variety of organisms from yeast to mammals [1–3]. In fact it is one of the 20 most abundantly expressed proteins in the cell. TCTP was initially identified in an Ehrlich ascites tumor cell line, hence the name [4]. Subsequently, TCTP was demonstrated to be present in almost all normal cells [5]. Despite the ubiquitous nature of TCTP, its exact cellular function is not clear and the true function of TCTP is still being debated. Interestingly, however, dysregulation of TCTP has been shown to be associated with several disease conditions such as cancer [6], Alzheimer's disease [7], and allergy [2, 8] suggesting an important role for TCTP in the physiological homeostasis of cells. Some of the other important cellular functions attributed to TCTP include calcium binding [9, 10] tubulin binding [11, 12], and antiapoptotic function [13, 14].

Several lines of studies suggest that expression of TCTP is upregulated by a variety of stress conditions such as oxidative stress, heat shock, and exposure to heavy metals. For example, TCTP is upregulated over 12-fold in hypoxic HeLa cells [15] and HepG-2 cells [16]. Similarly, oxidative stress induced by DTT and hydrogen peroxide (H_2_O_2_) can induce highly elevated levels of TCTP expression in yeast cells [1] and in human lung epithelial cells [17]. Our recent studies showed that TCTP can function as a potent antioxidant protein [18] in addition to chaperone-like activity [19] in the cell.

TCTP is a heat stable protein present mainly in the cytoplasm [5]. However, recent findings show that oxidative stress [20] and glucose stimulus [21] translocate TCTP to the nucleus. In addition, treatments with certain allochimeric molecules also result in the enrichment of TCTP in the nucleus [22]. Therefore, it is intriguing how TCTP gets transported into the nucleus. One of the ways by which proteins enter the nucleus involves binding to small molecular weight transporter proteins, such as SUMO, which covalently modifies (sumoylation) the protein [23]. Such covalent modification of cellular proteins by SUMO plays a central role in regulating a variety of cellular processes including nuclear transport, formation of subnuclear structures, regulation of transcriptional activities, DNA binding abilities of transcription factors, signal transduction, stress responses, cell-cycle progression, protein-protein interactions, and protein stability [24]. At present we do not know the clear function of TCTP in the nucleus. Given that TCTP might function as an antioxidant protein [18, 19], in the present study we asked the question whether sumoylation of TCTP has any significant role in the nuclear transport and protecting cell from damage during oxidative stress.

## 2. Materials and Methods

### 2.1. Sequence Analysis

TCTP sequence cloned from human bone eosinophilic granuloma cell line was analyzed to determine whether it contains any Small Ubiquitin-like Modifier (SUMO) motifs using SUMOplot software available in ExPASy proteomic tools.

### 2.2. Evaluating the Expression of TCTP in the Cytoplasm and Nucleus of Mammalian Cells

Human bone eosinophilic granuloma cell line (CRL-7802) obtained from ATCC (Manassas, VA) was cultured in DMEM supplemented with 10% FBS medium until it reached 100% confluence. Cytoplasmic and nuclear fractions were prepared using a kit purchased from Active Motif (Carlsbad, CA). Briefly, cells were washed once with 5 mL of ice-cold phosphate-buffered saline containing phosphatase inhibitors before removing them from the plate with Trypsin-EDTA. Approximately 1 × 10^6^ cells were resuspended in 500 *μ*L 1x hypotonic buffer and incubated on ice for 15 min. 25 *μ*L detergent was then added and vortexed for 10 seconds followed by centrifugation at 12000 rpm for 30 seconds at 4°C. The supernatant (cytoplasmic fraction) was stored at −80°C until use. The pellet was resuspended in 50 *μ*L of complete lysis buffer and vortexed for 10 seconds followed by incubating on ice for 30 min. The suspension was then centrifuged at 12000 rpm for 10 minutes at 4°C. The supernatant containing nuclear fraction was stored at −80°C until use. The protein concentrations of cytoplasmic and nuclear fractions were determined using a BCA kit (Thermo Fisher Scientific, Rockford, IL). Levels of TCTP expression in cytoplasmic and nuclear fractions were determined by western blot using polyclonal anti-TCTP purchased from MBL laboratories (Nagoya, Japan).

### 2.3. Detecting the Presence of Sumoylated TCTP in the Cellular Fractions

TCTP was immunoprecipitated using Seize X protein A immunoprecipitation kit obtained from Thermo Scientific Pierce Biotechnology (Cat.No. 45215). Briefly, 1 mg/mL of rabbit anti-TCTP antibody or preimmune rabbit serum was first allowed to bind to immobilized protein A columns for 15 minutes and washed with binding/wash buffer for 5 times in a microcentrifuge at 3500 rpm for 1 minute. In some studies, anti-FLAG monoclonal antibodies (Sigma, St. Louis, MO) were used to immunoprecipitate FLAG-labeled TCTP from cell preparations. Antibodies were first crosslinked to protein A using a DSS cross-linker before using in immunoprecipitation. For immunoprecipitation, 50 *μ*g of cytoplasmic and nuclear fractions were incubated with the crosslinked antibodies for 1 hour at room temperature. The unbound proteins were removed by washing five times with wash buffer (Thermo Fisher Scientific). The bound proteins were eluted with 200 *μ*L of immunopure elution buffer (Thermo Fisher Scientific). Eluted samples were resolved on 12% SDS-PAGE and transferred to nitrocellulose membrane and probed with 1 : 1000 rabbit polyclonal anti-SUMO antibodies (Imgenex, San Diego, CA) for 1 hour at room temperature. After washing, the membrane was incubated with 1 : 5000 goat anti-rabbit IgG coupled to HRP for 30 minutes at room temperature and signal developed using an ECL kit (Amersham, Pharmacia Biotech. Piscataway, NJ).

### 2.4. TCTP-Ubc9 GST Pull-Down Assay

Ubc9 gene cloned in pGEX-5X-1 vector at SmaI and BamHI sites was obtained from Dr. V. G. Wilson (Department of Medical Microbiology and Immunology, Texas A&M University System Health Science Center, College Station, TX). Both GST-Ubc9 fusion protein and GST alone were expressed in BL21 (DE3) and purified by glutathione-sepharose columns (Clontech, Palo Alto, CA). His-tagged-TCTP was expressed in BL21 (DE3) carrying pLysS plasmid and purified by cobalt metal affinity chromatography (Clontech). For the binding assay, approximately 2 *μ*g of GST alone or GST-Ubc9 fusion proteins was prebound to glutathione-sepharose beads by incubation for 1 hour at room temperature in 0.5 mL binding buffer (10 mM Tris-HCl, pH 7.4, 50 mM NaCl, 2% bovine serum albumin). Two *μ*g of purified TCTP was then added, and incubation was continued for 1 hour. The beads were washed five times with wash buffer (10 mM Tris-HCl, pH 8, 140 mM NaCl and 0.025% NaN_3_). Bound proteins were recovered by eluting with glutathione and were analyzed by western blotting using monoclonal anti-His tag antibody (Qiagen, Valencia, CA). Signals were detected using an ECL substrate kit (Amersham) and signal intensity quantitated using NIH image software.

### 2.5. *In Vitro* SUMO-1 Conjugation Assay

In order to test SUMO-1 modification of TCTP, HeLa cell extract containing SUMO-1 activating enzymes, UBA2/AOS1 was prepared as described previously [25]. Briefly, five *μ*g of purified His-tagged TCTP protein was incubated with 5 *μ*g of HeLa cell extracts in a 100 *μ*L reaction, containing an ATP-regenerating buffer (50 mM Tris-HCl, pH 7.6, 5 mM MgCl_2_, 2 mm ATP, 10 mM creatine phosphate, 3 units/mL creatine kinase, and 0.5 unit/mL inorganic pyrophosphatase), 6 *μ*g of purified SUMO-1, and 1 *μ*g of ubc9. Reactions were incubated at 37°C for 2 hours. Control reactions had one or more of the components omitted and replaced by additional buffer. The reaction was terminated by the addition of SDS-sample buffer and the reaction products were analyzed by western blotting with monoclonal anti-His tag antibody (Qiagen) by ECL method.

### 2.6. Construction of Wild-Type TCTP and Mutant TCTP Expression Vectors

The open reading frame (ORF) of wild-type TCTP from human bone eosinophilic granuloma was cloned into a pFLAG vector (Sigma). The forward PCR primer corresponded to the beginning of ORF of TCTP with the addition of an upstream in-frame *HindIII* restriction site (5′CCCAAGCTTATGATTATCTACCGGGACCTC3′). The reverse primer corresponded to the 3′ end of TCTP ORF flanked by *BamHI* restriction site (5′CGCGGATCCTTAACATTTTTCCATTTTTAA3′). PCR parameters were 95°C of denaturation for 30 seconds, 55°C of primer annealing for 30 seconds, 72°C of primer extension for 30 seconds, and the cycle was repeated for 30 times. A final extension of 5 minutes was performed at 72°C before storing the samples at 4°C. PCR products obtained were digested with HindIII and BamHI enzymes and ligated to similarly digested pFLAG mammalian expression vector. Mutant TCTP (Lys at aa164 mutated to Arg) was prepared using site-directed mutagenesis kit purchased from Stratagene (La Jolla, CA). Primer 1 corresponded to nucleotide 487-507 (TTTAGGGATGGTTTAAAAATG) of TCTP ORF to revert A residue at 491 to the G residue and a Sca I restriction site (5′AGTACT3′) was added upstream to primer 1. Primer 2 corresponded to nucleotide 466-486 (5′GAAAATCATATATGGGGTCAC′) of TCTP ORF. PCR parameters were 94°C for 4 min, 50°C for 2 minutes, and 72°C for 2 minutes. Followed by 8 cycles of 94°C for 1 minute, 56°C for 2 minutes, and 72°C for 1 minute. A final extension of 5 minutes was performed at 72°C. Following PCR, standard digestion, polishing and ligation was performed as recommended by manufacturer's protocol. Sequencing of both the DNA strands was done to confirm the authenticity of the wild-type and mutant TCTP sequences cloned in pFLAG vector.

### 2.7. Expression of Wild-Type and Mutant TCTP Constructs in Cos1 Cell Line

Cos1 cells purchased from ATCC were cultured in either 6- or 96-well tissue culture plates until they reached ~90% confluence. Cells were then transfected with pFLAG-TCTP or pFLAG-mutant TCTP using Lipofectamine 2000 or Oligofectamine transfection reagent (Invitrogen, Carlsbad, CA) as per the manufacturer's instructions. After 48 hours following transfection, cells were collected to prepare the cytoplasmic and nuclear fractions as described above. Expression of Flag-tagged constructs in these preparations was analyzed by western blotting with 1 : 1000 mouse anti-Flag monoclonal antibodies conjugated to HRP (Sigma). The blots were developed using an ECL method (Amersham).

### 2.8. Small Interfering RNA *In Vivo* Gene Silencing Assay

The siRNAs of TCTP and laminin A/C were synthesized at Dharmacon Research Inc. (Lafayette, CO). The target sequence of TCTP used for designing siRNA was from nucleotide 121-141 (5′AAGGTAACATTGATGACTCGC3′; sense siRNA, 5′-GGUAA CAUUGAUGACUCGCdTdT-3′; antisense siRNA, 5′-GCGAGUCAUCAAUGUUAC CdTdT-3′). For lamin A/C the target sequence (cDNA) was 5′-CTGGACTTCCAGAAGAACA-3′; sense siRNA, 5′CUGGACUUCCAGAAGAACAd Td T3′; antisense siRNA, 3′GACCUG AAGGUCUUCUUGU-5′. All procedures were performed under RNAse-free environment, using RNAse-free water. Approximately, 10^4^ cells/mL of human eosinophilic granuloma cells placed in 24-well plates were transfected with a final concentration of 50 nM of siRNA duplexes using Oligofectamine reagent as described above. Seventy-two hours after the transfection, cells were collected, lysed by addition of 100 *μ*L of SDS gel loading buffer, and subjected to SDS-PAGE and western blot analysis, using anti-Lamin A/C (Santa Cruz Biotechnology, Inc.) and anti-TCTP (MBL) antibodies.

### 2.9. H_2_O_2_ Tolerance Bioassay

Effect of oxidative stress in Cos1 cells was measured as described previously by Kuner et al. [26]. Briefly, Cos1 cells transfected with pFLAG-TCTP, pFLAG-mutant TCTP or human granuloma cells transfected with RNAiTCTP, or RNAiLamin were incubated with 100 *μ*M H_2_O_2_ for 24 hours in culture. Following incubation, the number of live cells was determined using a cell counting kit purchased from Dojindo Molecular Technologies (Gaithersburg, MA). Briefly, 10 *μ*L of the tetrazolium salt dye was added to the cells cultured in 96-well tissue culture plates and incubated for 4 hours. After incubation, the absorbance was measured at 450 nm using a microplate reader.

### 2.10. Statistical Analysis

Statistical analysis was performed using a Mann-Whitney *U* rank sum tests using SigmaStat 2.0 (Jandel Scientific Software, San Rafael, CA).

## 3. Results

### 3.1. Sequence Analysis of TCTP

Sequence analysis showed that TCTP cloned from human bone eosinophilic granuloma cells has a putative SUMO motif at aa 163-166. A comparison of the TCTP sequences from other species of organisms also showed the presence of putative SUMO motif. Predicted SUMO motifs in the TCTP sequence of other organisms are listed below: mouse TCTP (aa 163-166), rabbit TCTP (aa 163-166), yeast TCTP (aa 159-162), *Schistosoma mansoni* TCTP (aa 161-164), *S. japonicum* TCTP (aa 161-164), *S. haematobium* TCTP (aa 121-124), *Brugia malayi* TCTP (aa 107-110), *Wuchereria bancrofti* TCTP (aa 107-110), and *Onchocerca volvulus* TCTP (aa 107-110).

### 3.2. TCTP Binds to Ubc9 *In Vitro*

**Figure 1:**
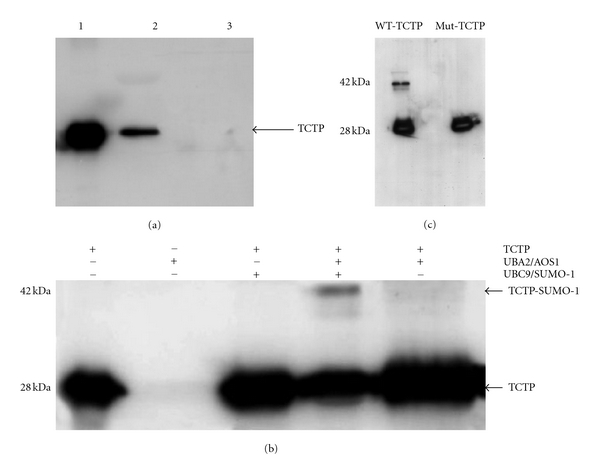
TCTP can be sumoylated *in vitro*. GST Pull-Down assay (a). Proteins were resolved on a 12% SDS-PAGE and transferred to a nitrocellulose membrane and probed with anti-HIS antibody. Lane 1: 5 *μ*g of rTCTP, Lane 2, shows the amount of TCTP that bound to the GST-Ubc9 enzyme, and Lane 3 shows the amount of TCTP that bound to GST alone control. These results show that TCTP strongly binds to Ubc9 enzyme. *In vitro* sumoylation assay (b). Proteins were resolved on 12% SDS-PAGE and transferred to nitrocellulose membrane and probed with anti-His or anti-TCTP antibody to detect TCTP. Note the high molecular weight product (TCTP-SUMO-1) in Lane 4, where the incubation mixture contained TCTP, UBA2/AOS1, and Ubc9/SUMO. Deletion of any one of these products from the reaction mixture resulted in the absence of this high molecular weight band (Lanes 1, 2, 3, and 5). Mutation studies (c), The lysine residue (aa 164) in the TCTP was mutated to arginine. When such mutated TCTP was subjected to *in vitro* sumoylation, SUMO-1 failed to bind to mutated TCTP, whereas wild-type TCTP could be sumoylated under the same conditions.

In order for TCTP to be sumoylated, an enzyme called Ubc-9 will have to bind to TCTP first which then catalyzes the binding of TCTP to SUMO-1 [25–28]. To determine whether the sumoylation motif in TCTP is active, we first performed GST Pull-Down assays using bacterially expressed GST-Ubc9. His-tagged TCTP purified by metal affinity column was mixed with GST-Ubc9 and affinity purified in GST column. Ubc9-bound protein was then probed with anti-His antibodies. Our results show that approximately 20% of the TCTP that passed through the column bound to GST-Ubc9 suggesting that TCTP can bind to Ubc9, an enzyme critical for sumoylation (Figure 1(a)). A similar Pull-Down assay with GST alone did not bind TCTP confirming that the binding of TCTP to Ubc-9 is specific.

### 3.3. SUMO-1 Is Covalently Conjugated to TCTP *InVitro*

Ubc9 was recently shown to act as an E2-conjugating enzyme that conjugates SUMO-1 to target proteins instead of ubiquitin [27, 28]. Prosite scan analysis showed that TCTP proteins have either one or two potential sites, where SUMO-1 can be conjugated. To test whether TCTP is a substrate for SUMO-1 modification by Ubc9, we used an *in vitro* reaction system described previously [24]. Following the reaction, a western blotting analysis was performed using anti-TCTP antibodies (Figure 1(b)) or anti-His antibodies (data not shown) to identify TCTP in the reaction mixture. Binding of SUMO-1 to TCTP increases the molecular mass and hence the sumoylated TCTP (~42 kDa) will migrate slower compared to native TCTP (~28 kDa) (Lane 4, Figure 1(b)). The higher molecular weight TCTP appeared only in the presence of SUMO-1, Ubc9, and the SUMO activating enzymes. Leaving out of any component in the reaction mixture failed to produce the high molecular weight TCTP (Figure 1(b)) suggesting that TCTP is a substrate for SUMO-1 conjugation that is mediated by Ubc9. Mutated TCTP (K164R) could not be sumoylated *in vitro*; however, the wild-type TCTP under the same condition was easily sumoylated (Figure 1(c)). These findings thus suggested that the lysine residue (Lys 164) in the SUMO motif of TCTP is critical for its sumoylation. Overall these findings confirmed that TCTP can be sumoylated.

### 3.4. TCTP Is Present in the Nucleus and Is Sumoylated

**Figure 2:**
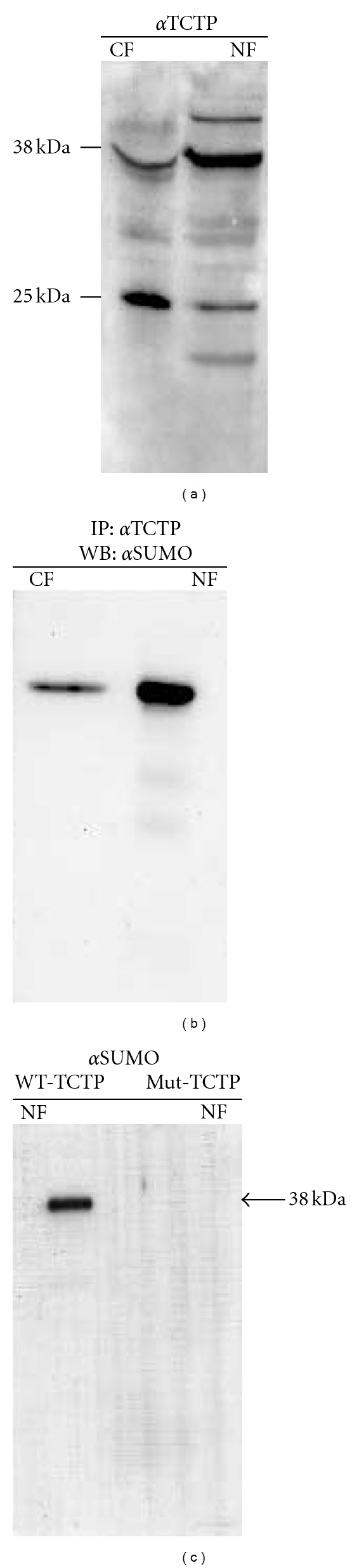
TCTP present in the nucleus is sumoylated. Lanes 1 and 2 show the proteins in the cytoplasm (CF) and nuclear (NF) fractions probed with an anti-TCTP antibody (a). The TCTP was then immunoprecipitated from these fractions using the anti-TCTP antibodies and the precipitate was subsequently probed with an anti-SUMO antibody (b). Lanes 3 and 4 show that sumoylated TCTP is present in both cytoplasm and nucleus. In order to determine if sumoylation is critical for the transport of TCTP into the nucleus, we used a FLAG-tagged TCTP to monitor the movement of TCTP inside the cell. Cos1 cells were transfected with either FLAG-tagged wild-type TCTP (W) or FLAG-tagged mutated TCTP (M). To determine whether the FLAG-tagged TCTPs were sumoylated, an anti-FLAG monoclonal antibody was used to immunoprecipitate the FLAG-tagged TCTP from the cytoplasm and nuclear fractions of the Cos1 cells. The bound TCTPs were then eluted, separated on a 12% gel, transferred to a nitrocellulose sheet, and probed with polyclonal anti-SUMO antibodies (c). These studies confirm that the high molecular weight form of wild-type TCTP present in the nuclear fraction is sumoylated. Results presented are representative of three similar experiments.

Analysis of the cytoplasm and nuclear fractions of the human eosinophilic granuloma cell line shows that TCTP is present in both cytoplasm and nucleus (Figure 2(a)). A western blot analysis with anti-TCTP antibodies recognized two species of TCTP, a 25 kDa protein and a 39 kDa protein in both the cytoplasm and nuclear fractions. Interestingly, the 39 kDa species was more abundant in the nucleus compared to the cytoplasm. To determine if TCTP is sumoylated, we first immunoprecipitated the TCTP from both cytoplasm and nuclear fractions, ran it on a gel, and probed with anti-SUMO antibodies. Our results confirm that the 39 kDa TCTPs present in the cytoplasm and nuclear fractions were sumoylated (Figure 2(b)). As expected only the high molecular weight form of TCTP was sumoylated and higher amounts of sumoylated TCTPs were present in the nucleus compared to the cytoplasm fraction (Figure 2(b)).

In the next series of experiments, we wanted to confirm that sumoylation is important for transport of TCTP into the nucleus. In these studies we used Cos1 cells transfected with FLAG-tagged TCTP. The flag helps us to identify and purify TCTP. In Figure 2(c) we show that we can demonstrate the presence of TCTP in the nuclear fraction of Cos1 cells. However, when we transfect Cos1 cells with mutated (K164R) FLAG-tagged TCTP, they fail to enter the nucleus (Figure 2(c)) and remain only in the cytoplasm. These studies confirm that sumoylation is critical for the transport of TCTP into the nucleus.

### 3.5. TCTP Can Partially Rescue the Cells from H_2_O_2_ Damage

**Figure 3:**
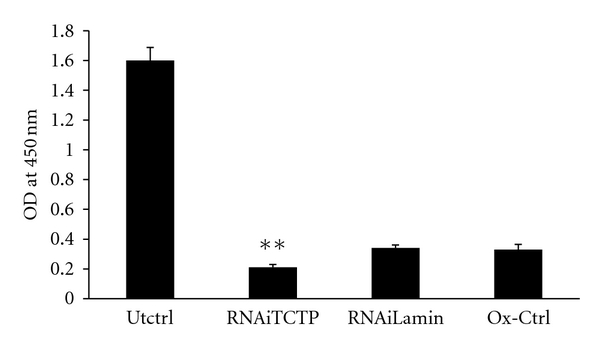
Knock down of TCTP by siRNA sensitizes cells to oxidative stress. Knocking down the wild-type TCTP with siRNA (RNAiTCTP) before exposing the cells to H_2_O_2_ significantly reduced the viability of human granuloma cells. Cells transfected with lamin SiRNA (RNAiLamin) or nontransfected cells exposed to the same conditions (O*_x_*-Ctrl) or cells without exposure to H_2_O_2_ (Utctrl) served as controls. Data presented is average of 3 wells and is representative of three similar experiments. **P < 0.05 compared to control siRNA (RNAiLamin) transfected group.

When human granuloma cells were exposed to oxidative stress, there was a significant reduction in the viability of the cells (Figure 3). Knocking down of the TCTP within the cells with siRNA further reduced the viability of the cells compared to cells mock-transfected with Lamin (Figure 3). These results suggest that TCTP within the cells can partially rescue cells from oxidative stress.

### 3.6. Overexpression of TCTP Conferred Resistance to H_2_O_2_ Damage

**Figure 4:**
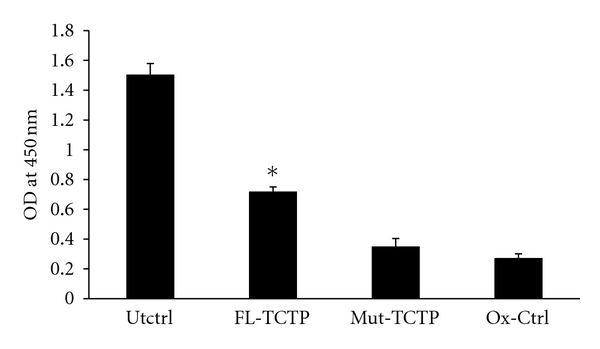
Over expression of TCTP conferred resistance to H_2_O_2_ damage in mammalian cells. Cos1 cells were over expressed with wild type (FL-TCTP) or mutant TCTP (Mut-TCTP) and exposed to 100 *μ*M H_2_O_2_ for 24 h in culture. Following incubation, cell viability was determined colorimetrically. Cells transfected with vector alone and exposed to the same conditions (O*_x_*-Ctrl) or without exposure to H_2_O_2_ (Utctrl) served as controls. Results show that mutation of TCTP at the SUMO motif significantly reduced its antioxidant function. *P < 0.01 compared to controls.

Cos1 cells overexpressed with TCTP were less susceptible to the damaging effects of oxidative stress induced by H_2_O_2_ (Figure 4). These results confirm our previous findings that TCTP has antioxidant function and can protect the cells from oxidative stress. To test if entry of TCTP into the nucleus is critical for its antioxidant function, we transfected Cos1 cells with TCTP mutated (K164R) in their SUMO motif (Mut-TCTP). When these cells were exposed to H_2_O_2_, there was a significant reduction in the viability of the cells (Figure 4). These findings thus suggest that entry of TCTP into the nucleus is critical for its antioxidant function.

## 4. Discussion

Several potential functions have been suggested for TCTP ranging from histamine release to putative antiapoptotic function. Nevertheless, a critical role for TCTP family of proteins within the cell remains to be fully described [29]. One of our recent studies showed that expression of TCTP is increased severalfold during oxidative stress and we demonstrated that TCTP can function as an antioxidant protein and as a chaperon [18, 19]. Studies by Lucibello et al. [30] show that TCTP plays a critical role in the survival of cancer cells during oxidative stress, reiterating our earlier findings that TCTP is a critical cytoprotective protein. Results presented in this study confirm that TCTP has antioxidant function. Our study also demonstrates that entry of TCTP into the nucleus is important for this antioxidant function. It is well established that TCTP is present in the nucleus. However, the mechanism of its entry into the nucleus has not been identified. In this study we show that TCTP transport into the nucleus is mediated by sumoylation.

TCTP is one of the 20 most abundantly expressed proteins within the cell [5, 6]. In fact, TCTP appears to be more abundant (approximately 100,000 copies per cell) than actin (approximately 60,000 copies/cell) in the yeast cell [31]. Majority of the TCTP proteins are present in the cytoplasm [5, 32]. However, several recent studies suggest that TCTP is also found abundantly in the nucleus, especially in certain cancer cells [13] and in cells undergoing mitosis [33] or stress [1]. It is not known how TCTP enters the nucleus. A sequence analysis of TCTP family of proteins suggested that they lack the obvious nuclear bipartite localization signals. So there has to be other mechanisms by which they enter the nucleus. Sumoylation plays a significant role in transporting proteins into the nucleus, especially proteins that lack nuclear bipartite localization signals [24, 34]. However, in order for a target protein to be transported, it should have appropriate motif for the binding of transporter protein. Analysis of the TCTP sequence using PROSITE scan tool showed that TCTPs possess potential SUMO motif with significant probability for sumoylation.

Using immunoprecipitation studies we were able to demonstrate that sumoylated form of TCTP can be found in both cytoplasm and nucleus of mammalian cells. In order for SUMO to bind to its target protein, it requires the help of an E2-conjugating enzyme, Ubc9 [25], that ligates SUMO to its target protein. The bound SUMO then covalently modifies the target protein, a process called sumoylation. In the absence of Ubc9 enzyme, the proteins cannot be sumoylated.* In vitro* functional assays in this study that measure the binding of Ubc9 and SUMO to TCTP showed that in the absence of Ubc9 enzyme TCTP was not sumoylated. Similarly, introducing a mutation in the SUMO motif (K164R) also prevented sumoylation of TCTP. Sumoylated form of TCTP is found both in cytoplasm and nucleus. When we mutated the SUMO motif in TCTP the mutated protein failed to enter the nucleus. These findings demonstrated that sumoylation of TCTP is potentially a critical event in the transport of TCTP into the nucleus. We did not do mass spectrometry or confocal microscopy to trace the movement of fluorescent-labeled TCTP from cytoplasm to nucleus. These additional techniques could have provided additional confirmatory evidence that sumoylation is critical for the transport of TCTP into the nucleus.

We previously reported that TCTP is an antioxidant protein [18] and can also function as a heat shock protein with chaperon-like activity [19]. In fact TCTP is found upregulated in various stress conditions [1] especially; the levels of TCTP are upregulated severalfold within minutes under oxidative stress [8, 15, 16]. Interestingly, SUMO and its conjugates are also increased during stress conditions [34]. Therefore, we hypothesized that sumoylation of TCTP is critical for its antioxidant function in the nucleus. Our results confirm that when we prevented TCTP from entering the nucleus by mutating SUMO motif, there was a significant reduction in the ability of TCTP to function as an antioxidant protein. The process of ubiquitylation tags the proteins for degradation, whereas sumoylation enhances their stability or modulates their subcellular compartmentalization for cell survival [27]. Thus, up regulation and entry of TCTP into the nucleus may be an important event in protecting the nuclear components and rescuing the cell from oxidative damages.

The present study thus reveals an important but hitherto unknown mechanism by which human TCTP enters the nucleus and functions as an antioxidant protein within the nucleus. Results presented in this study also suggest that sumoylation of TCTP is one such important mechanism for its nuclear transport at least in eosinophilic granuloma cells. Other redundant nuclear transport mechanism may be present for TCTP in other cell types and possibly may vary with the nature of the stress encountered. We identified one such mechanism or possibly the only mechanism of nuclear transport of TCTP. Presence of abundant amounts of TCTP inside the nucleus suggests that this ubiquitous molecule may have potential role in protecting the DNA and the cell from oxidative stress. These studies thus reveal an important cellular function for TCTP.

## Abbreviations

TCTP: Translationally controlled tumor proteins;
SUMO: Small ubiquitin like modifier protein;
UBC9: Ubiquitin carrier protein 9.
